# Persistent inequalities in 90-day colon cancer mortality: an English cohort study

**DOI:** 10.1038/bjc.2017.295

**Published:** 2017-08-31

**Authors:** H Fowler, A Belot, E N Njagi, M A Luque-Fernandez, C Maringe, M Quaresma, M Kajiwara, B Rachet

**Affiliations:** 1 Cancer Survival Group, Department of Non-Communicable Disease Epidemiology, Faculty of Epidemiology and Population Health, London School of Hygiene and Tropical Medicine, Keppel Street, London WC1E 7HT, UK

**Keywords:** short-term mortality, inequalities, colon cancer, socio-economic status, stage at diagnosis, comorbidity

## Abstract

**Background:**

Variation in colon cancer mortality occurring shortly after diagnosis is widely reported between socio-economic status (SES) groups: we investigated the role of different prognostic factors in explaining variation in 90-day mortality.

**Methods:**

National cancer registry data were linked with national clinical audit data and Hospital Episode Statistics records for 69 769 adults diagnosed with colon cancer in England between January 2010 and March 2013. By gender, logistic regression was used to estimate the effects of SES, age and stage at diagnosis, comorbidity and surgical treatment on probability of death within 90 days from diagnosis. Multiple imputations accounted for missing stage. We predicted conditional probabilities by prognostic factor patterns and estimated the effect of SES (deprivation) from the difference between deprivation-specific average predicted probabilities.

**Results:**

Ninety-day probability of death rose with increasing deprivation, even after accounting for the main prognostic factors. When setting the deprivation level to the least deprived group for all patients and keeping all other prognostic factors as observed, the differences between deprivation-specific averaged predicted probabilities of death were greatly reduced but persisted. Additional analysis suggested stage and treatment as potential contributors towards some of these inequalities.

**Conclusions:**

Further examination of delayed diagnosis, access to treatment and post-operative care by deprivation group may provide additional insights into understanding deprivation disparities in mortality.

Colon cancer, the fourth most frequently diagnosed cancer in England, has a less favourable 1-year prognosis in England when compared with some other common cancers, such as cancers of the rectum and breast (Exarchakou *et al*, 2015). One-year net survival for colon cancer (i.e., the survival of colon cancer if the other causes of death have been removed) was recently reported at around 75%, while it was largely >80% for the other cancers mentioned above (Exarchakou *et al*, 2015). Meanwhile, wide socio-economic inequalities in survival from colon cancer have been repeatedly reported in England (Mitry *et al*, 2008; Rachet *et al*, 2010), with worse prognosis for more deprived patients. These inequalities are generally evident shortly after diagnosis (Møller *et al*, 2012), with two-thirds of cancer deaths related to these inequalities occurring within 6 months after diagnosis (Ellis *et al*, 2012). Socio-economic inequalities in colon cancer survival have been reported in several countries (Wrigley *et al*, 2002; Aarts *et al*, 2010; Cavalli-Bjorkman *et al*, 2011; Ito *et al*, 2014) but have not been found in others (Dejardin *et al*, 2013; Antunes *et al*, 2016), which underlines the importance of understanding factors contributing to inequalities and more particularly differential short-term mortality in colon cancer patients in England.

Many published studies have focussed their attention exclusively towards subgroups of colon cancer patients, such as postoperative patients (Morris *et al*, 2011) or elderly postoperative patients (Dekker *et al*, 2011), to explain differences in short-term survival. A variety of indicators has been considered in studies examining SES – from metrics such as patient occupation or education level to deprivation indices. Common themes in the literature include examining the relationship between age, stage at diagnosis and short-term mortality (Gatta *et al*, 1998; Møller *et al*, 2012) and the relationship between cancer treatment and short-term mortality (Faivre *et al*, 2007). However, there was little discussion offered regarding the relationship between socio-economic status and risk factors for short-term mortality, such as comorbidity or stage at diagnosis. Awareness of factors contributing to different prognoses for short-term survival across all patient populations is central to the effective management of cancer care.

This study aims to quantify differences in 90-day mortality following diagnosis of colon cancer according to patients’ socio-economic status. We investigate to what extent these differences are influenced by age, stage at diagnosis, patient comorbidity score, whether the primary treatment received by the patient was a major surgery and whether the patient received major surgery as an elective or emergency procedure.

## Materials and methods

### Data

The analysis was undertaken on 69 769 adults aged 15–99 years diagnosed with colon cancer in England between 1 January 2010 and 31 March 2013 and followed up until 31 December 2014. The data for these analyses were obtained from the national cancer registry records (Office for National Statistics, 2014) linked with Hospital Episode Statistics (HES) records (Health and Social Care Information Centre, 2013) and national bowel cancer clinical audit data (Health and Social Care Information Centre, 2014a) – representing information collated from clinicians working in multidisciplinary teams who have direct involvement with the patients. The linkage of the registry data with clinical audit data and HES records was undertaken using an algorithm developed by the Cancer Survival Group at the London School of Hygiene and Tropical Medicine (Shack, 2009), which prioritised linkage of records according to the combination of patient identifier variables (Office for National Statistics, 2014). The cancer registry data represented >99% of cancer registrations in England (Office for National Statistics, 2014) and provided information on gender, age at diagnosis, socio-economic status, tumour stage and date of diagnosis. The clinical audit data had a case ascertainment of 94% (Health and Social Care Information Centre, 2014b) and captured information on tumour stage and treatment. To obtain the most robust information on stage at diagnosis from clinical audit and registry data, we used an algorithm (Benitez-Majano *et al*, 2016) that creates a composite stage at diagnosis variable, based on the rules of the Union for International Cancer Control TNM classification of malignant tumours. It combined available information in individual tumour (T), nodes (N) and metastases (M) stage components, prioritising information captured in the clinical audit data and only using registry stage data where this was not present, to derive a four-level ordinal stage variable, where stage 1 represents localised stage cancer and stage 4 indicates metastatic stage cancer (Benitez-Majano *et al*, 2016).

HES records were used to supplement treatment information gathered from the clinical audit data and to derive information on comorbidity prevalence. We devised an algorithm to derive the first major surgical treatment received by each patient within a time window of between 30 days prior and 90 days following cancer diagnosis. Treatment information was derived from data coded according to the Office of Population Censuses and Surveys (OPCS) Classification of Interventions and Procedures (fourth version, ‘OPCS-4’) (Health and Social Care Information Centre, 2017). The OPCS-4 codes represent an information standard used by clinical coders within National Health Service hospitals in Great Britain. In the clinical audit data, we used the sole OPCS-4-coded treatment variable describing the patient’s primary procedure. In HES up to 12 fields (among a total of 20) capturing OPCS-4-coded treatment information had been completed for each hospital episode. Major surgery was categorised using the definition of major treatment devised by the Site-Specific Clinical Reference Groups of the National Cancer Intelligence Network (National Cancer Intelligence Network, 2013), which sought extensive input from clinicians and oncologists. Supplementary Table 1 defines OPCS-4 codes representing major surgery for colon cancer. Surgery presentation was defined as either ‘emergency’ or ‘elective’, according to the method of admission recorded in HES.

Information on the presence of comorbidities and other conditions were derived from the historical records of diagnosis fields in HES between 2003 and 2013, allowing the capture of information up to 6.5 years prior to cancer diagnosis. Each hospital spell record can contain up to 20 diagnostic fields in which coexisting conditions could be recorded. We extracted information on the prevalence of the 17 comorbidities of the Charlson Index (Charlson *et al*, 1987) plus obesity by applying an algorithm (Maringe *et al*, 2017) that created a binary variable to flag the presence of each of the comorbidities of interest prior to the date of cancer diagnosis recorded in the registry data. We considered comorbidities recorded during a retrospective 6-year period from 0.5 to 6.5 years prior to cancer diagnosis in the analysis. Cancer registry data were the source of information for the two Charlson Index comorbidities relating to cancer (i.e., ‘any malignancy’ and ‘metastatic solid tumours’). However, stage information was largely missing for many cancers and, where available, we found only 11 colon cancer patients with a metastatic tumour (i.e., TNM stage 4 tumour) prior to the diagnosis of their colon cancer. We therefore considered any prior cancer diagnosis as ‘any malignancy’ in the context of the Charlson Index. Each patient’s Charlson Comorbidity Index score was calculated based on Charlson’s weighted index of comorbidity (Charlson *et al*, 1987). A weighted score was derived for each patient based on whether any of the comorbidities had been diagnosed, as confirmed from the binary comorbidity indicators. The Charlson Comorbidity Index scores were then summarised by creating a four-category comorbidity score variable, indicating whether the Charlson Comorbidity Index score was 0, 1, 2 or ⩾3.

We defined socio-economic status as deprivation, which was measured using the Income Domain from the 2011 England Indices of Multiple Deprivation (Department for Communities and Local Government, 2011) defined at the Lower Super Output Area level (mean population 1500). Patients were allocated to one of the five deprivation categories according to their area of residence at the time of their cancer diagnosis. This ecological, five-level ordinal variable represents a scale of deprivation, where ‘1’ represents the least deprived and ‘5’ represents the most deprived category of patients, based on the quintiles of the distribution areas in England.

### Analysis

All analyses were conducted using Stata 14 (StatCorp LP, College Station, TX, USA) (StataCorp, 2015).

Information on patient characteristics was investigated using cross tabulations to explore the distribution and completeness of variables of interest for each gender.

The first step was to quantify the role of key variables on the 90-day mortality, which then enabled us to derive a series of indicators with public health relevance, discussed in the next subsection. Prior to conducting our analyses, the relationships between the prognostic factors (age, stage at diagnosis, treatment received and comorbidity score), our primary exposure of interest (deprivation) and the outcome of interest (90-day mortality) were considered. A directed acyclic graph depicting the assumed relationships between these variables is provided in Supplementary Figure 1.

We used multivariable logistic regression models to estimate the associations between the probability of death occurring within 90 days of colon cancer diagnosis (our outcome of interest) and deprivation as well as age at diagnosis, stage at diagnosis, comorbidity score and treatment received, separately for each gender. The possible nonlinear effect of age at diagnosis was modelled using a quadratic regression spline with one knot at 70 years (near the mean age of the patients, 72.2 years). Furthermore, because the association between deprivation and 90-day mortality might vary by stage, comorbidity and treatment, the initial multiple logistic regression model included, in addition to the variables mentioned above, the corresponding interactions. Similarly, interactions between stage and age, stage and treatment, stage and comorbidity and comorbidity and treatment were initially considered on *a priori* clinical grounds. A backward elimination method (Agresti, 2013) was then applied to select the most parsimonious model to predict 90-day mortality. This final analysis model was used to predict our outcome – probability of death within 90 days of diagnosis. As a further step, to examine the contribution stage and treatment made towards this outcome, we performed additional analysis removing in turn from our final analysis model, stage, treatment and both stage and treatment.

Multiple imputations accounted for 30% missing composite stage, assuming a missing at random mechanism (Little and Rubin, 1987) – that is, that the probability of stage being missing depended on the observed data. Given that the logistic regression model included (i) interactions between stage and other variables (and stage had missing information), and (ii) a nonlinear effect of age, multiple imputation by the substantive-model compatible fully conditional specification (SMC–FCS) method (Bartlett *et al*, 2015; Bartlett and Morris, 2015) was employed to ensure compatibility between the imputation model and the analysis model (Carpenter and Kenward, 2013). Separately for each gender, a multinomial logistic regression model was used to impute stage, including as predictor variables, (i) all the variables in the analysis model mentioned above, (ii) the vital status within 90 days, (iii) the tumour grade (a four-level ordinal variable indicating the level of differentiation of the tumour), and (iv) a variable representing the Nelson–Aalen estimate (an estimator of the cumulative hazard of death) (Falcaro *et al*, 2015). In this model, age was also included as a nonlinear effect, to ensure compatibility (Carpenter and Kenward, 2013) with the analysis model. Tumour grade was used as an auxiliary variable in the imputation model, and as it also had missing information (20% missing grade), a multinomial logistic regression model was also used to impute it within the SMC–FCS framework. It is important to mention that ordinal logistic regression models, rather than multinomial logistic regression models, could be employed to impute stage and grade, provided that care is taken to test for the usually made proportional odds assumption (Agresti, 2013; Carpenter and Kenward, 2013).

The Stata ‘smcfcs’ command (StataCorp, 2015) was used to generate 30 imputed data sets, using the imputation model and imputation strategy above. The initial multiple logistic regression model was fitted to the 30 imputed data sets and Rubin’s Rules (Little and Rubin, 1987) used to combine the analysis results. The backward elimination model selection method was applied using the ‘mi test’ command (StataCorp, 2015) to perform a multivariate Wald test, dropping the most insignificant interaction term one at a time; refitting the reduced model to the data imputed as above and testing again for the remaining interactions, until all remaining interactions were significant (*P*-value⩽0.05). All interactions were tested on the same set of imputed data: retaining the data imputed using the most complex imputation model and testing all subsequent reduced models on these same set of data ensures valid estimation of all the reduced models (Carpenter and Kenward, 2013; Bartlett and Morris, 2015).

### Indicators produced

Using the final model selected for each gender, we predicted two sets of probabilities of dying within 90 days of diagnosis. The first consisted of conditional probabilities, that is, given specific values of the prognostic factors. This was performed using the ‘mi estimate’ command (StataCorp, 2015). The second set included average predicted probabilities estimated for each deprivation group in turn; we predicted the probability of death within 90 days of diagnosis for each patient, adjusting for prognostic factors, and averaged these probabilities on all patients in the subgroup (Muller and Maclehose, 2014). This meant the probability of death was predicted for each patient in the deprivation group – adjusting for the patient’s prognostic factor values – and used to derive the average predicted probability of death of all patients in that deprivation group. By way of comparison, the average predicted probabilities were then recalculated separately for each of the deprivation groups but setting the level of deprivation as the least deprived group and with all other prognostic factors remaining as observed. This gave an estimate of the probability of death as if patients had been in the least deprived group. The difference between those two probabilities quantified the effect of deprivation. This was performed using the ‘mimrgns’ command. In the additional analysis, we reiterated this calculation of average predicted probabilities as if patients had been in the least deprived group but using the final model without stage, then without treatment and finally without both.

## Results

A total of 69 769 adults were diagnosed with colon cancer in England between January 2010 and March 2013; 36 685 (52.6%) of these adults were males. Table 1 shows the characteristics of the patients in this study overall and for the 14.7 and 16.6% males and females, respectively, who died within 90 days after a colon cancer diagnosis. As deprivation increased the percentage of patients in each deprivation group decreased in both genders (22.3 in deprivation group 1 *versus* 15.2% in deprivation group 5 in males and 21.4 *versus* 15.3% in females). However, by deprivation group, the percentage of patients who died in 90 days increased with deprivation: in the least deprived group, the percentage was 12.4 in males and 13.3% in females; and in the most deprived group, the percentages were 17.7 and 19.9 in males and females, respectively. Both male and female elderly patients were highly represented, especially the 80+ patients, in the patients who died within 90 days. Stage at diagnosis was missing for almost a third of the patients but for 38.1% and 44.3% of males and females who died within 90 days, respectively. Early stages (1 and 2) represented a third of the patients with observed stage but about 13% of those who died within 90 days. Only 74 males and 54 females diagnosed with stage 1 died within 90 days, compared with >2000 diagnosed with the most advanced stage 4 (representing three-quarters of the patients who did not survive). The patients with no recorded comorbidity were about three-quarters of all patients but were two-thirds of those who died within 90 days, illustrating an overrepresentation of those with no recorded comorbidity among the group who died within 90 days. About one-third of the patients did not receive any major surgery, but this group with no treatment represented over three-quarters of those who died within 90 days. By contrast, <10% of the patients who did not survive received a major elective surgery while they represented nearly half of all patients. We provide the distribution of stage at diagnosis in patients receiving a major emergency, major elective surgery or no major surgery in Supplementary Table 2. Of the 14 810 patients with known tumour stage who did not have a major surgery, 11.1% had a stage 1 diagnosis, whereas 66.6% had a stage 4 diagnosis. Supplementary Table 3 shows the distribution of patients who underwent major surgery via either emergency or elective presentation – and those who did not have major surgery – by comorbidity score. The majority of patients (51%) with the highest comorbidity score of 3 did not undergo major surgery while 34% of these patients had a major elective surgery. By contrast, 49% of patients with no recorded comorbidity had a major elective surgery and 33% no major surgery.

In building the multivariable model for obtaining the predictions of probability of death within 90 days, the model selection strategy identified significant interactions in both male and female patients, that is, the association between age and 90-day mortality was modified by stage, comorbidity and treatment. There was also an interaction between treatment and stage. In males only, one additional interaction was retained between comorbidity score and treatment.

### Probability of death within 90 days of colon cancer diagnosis

#### Conditional probabilities by prognostic factor patterns

In both male and female patients, the conditional (i.e., conditional to specific values of factors) probability of death within 90 days rose with increasing level of deprivation, whatever the age and stage at diagnosis. To investigate this at a more granular level, the deprivation groups were split into subgroups of patients, according to the presence of comorbidities and the treatment they received. Therefore, for each sex, probability of death within 90 days was assessed in terms of each level of deprivation, age, stage, comorbidity score and treatment.

Table 2 presents the probability of death within 90 days for male and female patients in the most and least deprived patients, according to their age at diagnosis (60, 70 and 80 years), stage at diagnosis, comorbidity (no recorded comorbidity and the highest score of 3) and their treatment (major emergency surgery, major elective surgery or no major surgery). The most deprived patients were systematically more likely to die within 90 days than the least deprived patients, irrespective of age, stage, comorbidity and treatment status. For example, in males aged 70 years, with stage 2 diagnosis, comorbidity score 3 and who underwent a major emergency treatment, the probability of dying within 90 days was 11.7% (95% confidence interval (CI) 8.3, 16.3%) in the most deprived compared with 7.9% (95% CI 5.5, 11.2%) in the least deprived patients. Similarly, among those who underwent a major elective surgery, the probabilities of death were 3.8% (95% CI 2.7, 5.4%) and 2.5% (95% CI 1.7, 3.6%) in the most and least deprived, respectively. The probability of death varied by deprivation even among patients with no recorded comorbidity, regardless of their treatment. We observed very similar patterns in females.

**Figure 1:**
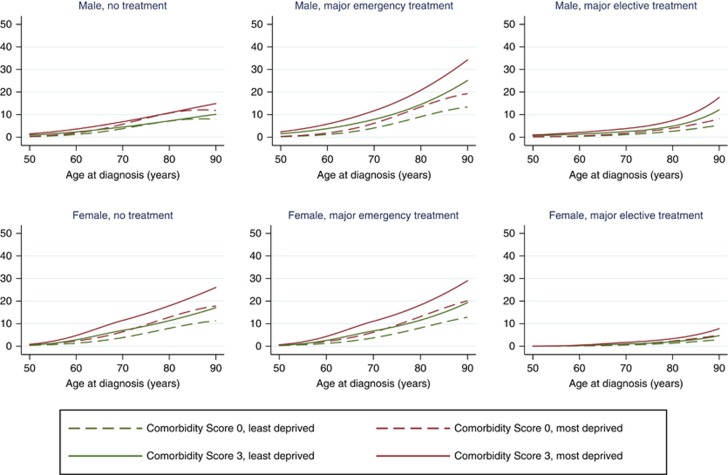
**Probability (%) of death within 90 days of stage 2 colon cancer diagnosis according to age at diagnosis, among patients with (i) comorbidity score of 0 or 3 and (ii) from the least or the most deprived group.**

By contrast, stage-2 patients with no recorded comorbidity experienced comparable 90-day mortality whether they had no major surgery or a major emergency surgery (Figure 1, Table 2). This was the case across all deprivation groups. For example, the probability of 90-day mortality among the most deprived female patients aged 70 years was 6.4% (95% CI 4.6, 8.8%) if not receiving any major surgery and 6.2% (95% CI 4.6, 8.3%) when receiving a major emergency surgery. These probabilities were much lower among females receiving a major elective surgery (1.0%, 95% CI 0.7, 1.6% with the same combination of factors). Among men only, the effect of comorbidity on the probability of 90-day mortality according to treatment differed: highest in those receiving emergency surgery, lowest in those receiving elective surgery, and intermediate in those with no surgery, regardless of their deprivation.

We provide graphs illustrating probability of death within 90 days of colon cancer diagnosis against age at diagnosis for each treatment type in both males and females, at each of the four stages of diagnosis (Figure 1, Supplementary Figures 2–4). The graphs contrast probability of death in the most and least deprived groups in patients with no recorded comorbidities and those with the maximum comorbidity score of 3. The general pattern was that more deprived patients were associated with higher 90-day mortality, regardless of the combination of other prognostic factors. Figure 1 presents these probabilities for stage-2 patients.

#### Average predicted probabilities by deprivation

In both males and females, the average predicted probabilities of death within 90 days of diagnosis identified a clear gradient across the observed deprivation groups, highest among the most deprived patients and lowest among the least deprived patients (Table 3, second and fourth columns). The existence of a difference in the average predicted probability of death between the most deprived and least deprived patient groups can be termed a ‘deprivation gap’. The largest deprivation gap was in females, with 6.5% fewer deaths predicted within 90 days for the least deprived group compared with the most deprived (least deprived: 13.4%, 95% CI 12.7, 14.0% and most deprived: 19.9%, 95% CI 19.0, 20.7%). In males, this gap was 5.3% (least deprived: 12.4%, 95% CI 11.8, 13.1% and most deprived: 17.7%, 95% CI 16.9, 18.6%).

To explore the deprivation gap further, the probability of death was recalculated for each of the deprivation groups by predicting each group’s probability of death as if it were the least deprived group, while all other prognostic factors remained as observed (Table 3, third and fifth columns). The results showed a shrinkage in the difference in probability of death between the most and least deprived groups, the difference became 1.7% and 1.3% in females and males, respectively. As expected, we observed the largest reduction in probability of death in the most deprived group: in females the reduction was 4.8% and in males the reduction was 4%.

In the additional analysis, when stage, treatment or both stage and treatment in combination were excluded from the final model and when the average predicted probability of death was calculated as if patients belonged to the least deprived group, there was a further reduction in the mortality difference between the most and least deprived patients from the one seen in the full analysis model (Supplementary Table 4). When stage alone was excluded from the model, the difference in probability of death between the most and least deprived patients reduced by approximately 30% (absolute difference of 0.9% compared with 1.3% with the final full model in males and 1.2% compared with 1.7% in females). For treatment alone, this percentage was 25% (absolute difference of 1% compared with 1.3% in males, and 1.3% compared with 1.7% in females). This suggested that, after accounting for the effect of deprivation, stage (or treatment) contributed to approximately one-third (or a quarter) of the remaining difference. When both stage and treatment were removed from the model, the remaining difference was close to 0%, indicating that in combination stage and treatment appear to contribute to most of the remaining difference in 90-day mortality once the effect of deprivation has been accounted for.

## Discussion

We found a very wide overall range of short-term mortality probabilities: for example, among female patients aged 60 years, those diagnosed with early stage colon cancer and who underwent a major elective surgery had a 90-day mortality probability near 0%, compared with a probability between 25% and 55% (depending on their deprivation group and comorbidity) for those with a stage-4 diagnosis who did not receive a major surgery. Deprivation differences in 90-day mortality were seen despite adjusting for major prognostic factors.

In our patient population, when calculating the average predicted probability of death within 90 days by deprivation group, and assuming that patients belong to the least deprived group, the differences between the probability of death in the least deprived group and the other deprivation groups became smaller. It translated to a total of 209 fewer deaths within 90 days of diagnosis per year in females and 168 fewer deaths per year in males (about 4% of the number of deaths observed in deprivation groups 2–5). Furthermore, when considering only the most deprived group of patients, it equated to approximately 74 and 69 fewer deaths in females and males, respectively. These results suggested that the differential distribution of the prognostic factors in deprivation groups may account for some of the outcome differences observed.

In the additional analysis, when we explored the role stage and treatment may be having in 90-day mortality differences between the most and least deprived patients, we found that stage and treatment appeared to contribute towards almost all of the remaining difference between the most and least deprived patient groups. When the average predicted probability of death was calculated assuming all patients were in the least deprived group and after removing both stage and treatment from the model, the average predicted probabilities were almost the same between the most and least deprived patient groups.

Differential access of treatment by deprivation may explain some of the inequalities in mortality. The percentage of colon cancer cases diagnosed during an emergency admission was as high as 31.4% in England for diagnoses made between 2006 and 2010 (Abel *et al*, 2015). In Scotland, the most deprived patients were more likely to present as an emergency and undergo palliative surgery (Oliphant *et al*, 2013). The same study also found higher postoperative mortality among more deprived colorectal patients, after adjusting for comorbidity and stage. These findings are in line with ours, where the socio-economic differences in 90-day mortality were evident in both nonoperative and postoperative colon cancer patients. Comorbidity and stage provide some contribution towards differences in short-term cancer mortality, but the socio-economic inequalities in 90-day mortality probability persisted even within any given treatment category, after controlling for stage and comorbidity. In the context of randomised clinical trials, where stage, comorbidity and also treatment are well controlled, we did not find evidence of a deprivation gap in 1-year colorectal cancer survival (Nur *et al*, 2008).

In a broader context, other work discussed differences in the quality of postoperative care and availability of beds in high dependence and intensive care units in the institution where treatment is received as potential factors influencing short-term postoperative mortality (Morris *et al*, 2011). This is especially pertinent in the presence of a high prevalence of postoperative complications. Previous research in Australia found that patients from higher socio-economic areas had a lower risk of developing postoperative complications (Beckmann *et al*, 2016), reinforcing the need for adequate postoperative care facilities in the most deprived areas. Comparing postoperative care resourcing between institutions in more and lesser deprived areas could provide some explanation behind socio-economic differences in 90-day colon cancer mortality and should be examined in more detail. Our study included surgical treatment received up to 90 days after diagnosis but did not account for the time to surgical treatment. Other research has shown more deprived patients were more likely to receive late treatment, that is, later than a month since diagnosis (Lejeune *et al*, 2010).

The results of this study provided some insight into an existing dynamic between treatment, comorbidity and short-term mortality. This relationship has some complexity, as comorbidity affects survival and influences cancer management (Faivre *et al*, 2007). This complexity is more pronounced in the elderly: the frequency of comorbidity is often higher in the elderly (Colorectal Cancer Collaborative Group, 2000), they tend to have more advanced disease stage (Dekker *et al*, 2011), and postoperative mortality increases with age (Morris *et al*, 2011). Additionally, patients undergoing emergency major surgery had a higher 90-day probability of death than patients who had received elective major treatment. This concurs with previous research indicating emergency surgery in comorbid patients to be a risk factor for short-term mortality in postoperative colon cancer patients (Gooiker *et al*, 2012).

Some challenges were faced when conducting analyses for this study, in particular owing to the use of population-based data. We employed a robust imputation strategy to mitigate the disadvantage of having missing information on stage (Bartlett *et al*, 2015). This approach allowed us to select the most parsimonious model as the final model to predict probability of death within 90 days, while taking proper account of the missing data. The overall probability of 90-day mortality was estimated as 14.7% (95% CI 14.4, 15.0%) for males and 16.6% (95% CI 16.3, 16.9%) for females. These predictions closely align with the observed proportion of deaths occurring within 90 days in our study population, confirming the goodness of fit of the analysis model on the observed data.

Information on treatment received by patients in our study was obtained using records from public hospitals. Some patients in our study may have received treatment in private facilities, which is not captured in our study. However, the proportion of cancer patients receiving surgical treatment outside the National Health Service has historically been small (Lawrence, 2013).

Our method for deriving patient comorbidity information was dependent on patients visiting the hospital and being diagnosed with the comorbidity in the time period of interest. We acknowledge the possibility that some comorbid adults may not have attended hospital nor had a comorbidity diagnosis recorded in this time, and therefore their comorbidity score would be 0. When available, including nonsurgical treatment in the analysis may provide additional insight into disparities in 90-day mortality between colon cancer patient groups, although surgery remains the primary curative treatment for colon cancer.

In conclusion, this study gives a full picture of 90-day probability of death according to the main prognostic factors and highlights persistent socio-economic inequalities in short-term mortality, even after accounting for the main prognostic factors, including prediagnosis comorbidities that could be derived from hospital attendance records and cancer registry data. Indeed, these socio-economic differences in 90-day mortality were especially apparent in the older patients, as probability of 90-day mortality increased with age. The provision of treatment involves consideration as to whether patients can withstand the trauma of surgery, particularly where patients have comorbid conditions. This is especially true in older patients more vulnerable to the aftermath of surgery. The planning of cancer treatment and care would need to focus decisions to treat patients on patient performance status and comorbidities, rather than their chronological age (Lawler *et al*, 2014). Socio-economic inequalities in 90-day mortality being found even among non-vulnerable patients suggest that resources for optimal treatment planning and postoperative care facilities may not be equally accessible to all patient deprivation groups. This study has identified a need to focus on understanding what is driving the effect of deprivation on 90-day mortality, including differences in health-care-seeking behaviours. Based on the findings of this population-based study, beneficial health policy initiatives could include targeted screening programmes to facilitate earlier-stage diagnosis in vulnerable patient groups, improved preoperative planning, including evaluation of comorbid patients, and more stringent postoperative monitoring of the patients.

## Figures and Tables

**Table 1:** Patient characteristics – patients diagnosed with colon cancer 2010–2013^a^ in England

	**Males**				**Females**			
	**Total**		**Died within 90 days**		**Total**		**Died within 90 days**	
	***N***	**%**	***n***	**%**^b^	***N***	**%**	***n***	**%**^b^
**Deprivation (Income Indices of Multiple Deprivation)**								
Least deprived (1)	8180	22.3	1018	18.9	7078	21.4	946	17.2
2	8231	22.4	1124	20.9	7241	21.9	1109	20.2
3	7629	20.8	1114	20.7	7048	21.3	1195	21.8
4	7072	19.3	1132	21.1	6649	20.1	1232	22.4
Most deprived (5)	5573	15.2	989	18.4	5068	15.3	1006	18.3
**Age (years)**								
15–40	643	1.8	29	0.5	736	2.2	20	0.4
41–50	1182	3.2	68	1.3	1202	3.6	50	0.9
51–60	3386	9.2	283	5.3	2966	9.0	232	4.2
61–70	9889	27.0	840	15.6	7226	21.8	638	11.6
71–80	12 171	33.2	1674	31.1	9785	29.6	1333	24.3
81–99	9414	25.7	2483	46.2	11169	33.8	3215	58.6
**Stage**								
Missing	11 186	30.5^c^	2049	38.1^c^	10 531	31.8^c^	2430	44.3^c^
1 (localised)	3355	13.2^d^	74	2.2^d^	2647	11.7^d^	54	1.8^d^
2	7084	27.8^d^	368	11.1^d^	6571	29.1^d^	338	11.1^d^
3	6667	26.1^d^	388	11.7^d^	6006	26.6^d^	401	13.1^d^
4 (metastatic)	8393	32.9^d^	2498	75.1^d^	7329	32.5^d^	2265	74.1^d^
**Comorbidity score**								
0	26 314	71.7	3380	62.9	24 982	75.5	3600	65.6
1	3780	10.3	697	13.0	3552	10.7	772	14.1
2	3558	9.7	631	11.7	2521	7.6	542	9.9
3+	3033	8.3	669	12.4	2029	6.1	574	10.5
**Treatment**								
No major treatment	13 390	36.5	4056	75.4	11 811	35.7	4290	78.2
Major emergency treatment	5749	15.7	794	14.8	5962	18.0	868	15.8
Major elective treatment	17 546	47.8	527	9.8	15 311	46.3	330	6.0
Total	36 685	100.0	5377	14.7	33 084	100.0	5488	16.6

a 2013 data represent diagnosis between 1 January 2013 and 31 March 2013.

b Representing the percentage of patients within each gender who died within 90 days.

c Representing the percentage of patients with missing stage information.

d Calculated as a percentage of patients with complete stage information.

**Table 2:** Conditional probabilities of death within 90 days of colon cancer diagnosis

			**Male**			**Female**		
			**Age at diagnosis, years**			**Age at diagnosis, years**		
			**60**	**70**	**80**	**60**	**70**	**80**
	**Comorbidity score**	**Deprivation group**	**PoD (%) (95% CI)**	**PoD (%) (95% CI)**	**PoD (%) (95% CI)**	**PoD (%) (95% CI)**	**PoD (%) (95% CI)**	**PoD (%) (95% CI)**
**Stage 1**								
No major treatment	0	Least	0.2 (0.1; 0.6)	0.7 (0.4; 1.3)	1.9 (1.2; 2.9)	1.4 (0.8; 2.3)	3.8 (2.7; 5.4)	8.0 (6.2; 10.2)
		Most	0.4 (0.1; 1.0)	1.1 (0.7; 2.0)	2.9 (1.8; 4.5)	2.3 (1.4; 3.9)	6.4 (4.6; 8.8)	12.9 (10.1; 16.2)
	3	Least	0.5 (0.2; 1.3)	0.9 (0.5; 1.6)	1.9 (1.2; 3.0)	2.8 (1.5; 5.5)	7.0 (4.7; 10.3)	11.3 (8.5; 14.9)
		Most	0.7 (0.3; 2.0)	1.4 (0.8; 2.4)	2.9 (1.8; 4.6)	4.8 (2.5; 9.0)	11.4 (7.8; 16.4)	17.9 (13.8; 22.9)
								
Major emergency treatment	0	Least	0.3 (0.1; 1.4)	1.2 (0.4; 3.8)	3.6 (1.2; 10.4)	1.2 (0.7; 2.1)	3.7 (2.7; 5.0)	8.1 (6.5; 10.0)
		Most	0.5 (0.1; 2.2)	1.8 (0.6; 5.8)	5.4 (1.8; 15.2)	2.1 (1.3; 3.5)	6.2 (4.6; 8.3)	13.1 (10.7; 16.0)
	3	Least	1.1 (0.3; 4.7)	2.4 (0.7; 7.6)	5.9 (1.9; 16.6)	2.6 (1.4; 4.9)	6.8 (4.7; 9.7)	11.6 (9.0; 14.8)
		Most	1.7 (0.4; 7.1)	3.6 (1.1; 11.3)	8.9 (3.0; 23.7)	4.3 (2.3; 8.0)	11.1 (7.8; 15.4)	18.3 (14.5; 22.8)
								
Major elective treatment	0	Least	0.2 (0.1; 0.5)	0.5 (0.3; 0.8)	1.5 (1.0; 2.3)	0.1 (0.1; 0.3)	0.5 (0.4; 0.8)	1.4 (1.0; 1.8)
		Most	0.3 (0.1; 0.7)	0.8 (0.5; 1.3)	2.4 (1.6; 3.5)	0.2 (0.1; 0.4)	0.9 (0.6; 1.3)	2.3 (1.8; 3.0)
	3	Least	0.6 (0.2; 1.8)	1.1 (0.6; 1.9)	2.9 (1.8; 4.5)	0.3 (0.1; 0.6)	1.0 (0.7; 1.6)	2.0 (1.5; 2.7)
		Most	1.0 (0.3; 2.7)	1.7 (1.0; 3.0)	4.4 (2.8; 6.7)	0.5 (0.2; 1.0)	1.7 (1.1; 2.6)	3.4 (2.5; 4.6)
**Stage 2**								
No major treatment	0	Least	1.2 (0.7; 1.9)	3.8 (2.8; 5.1)	7.1 (5.5; 9.3)	1.4 (0.8; 2.3)	3.8 (2.7; 5.4)	8.0 (6.2; 10.2)
		Most	1.9 (1.2; 3.0)	5.7 (4.2; 7.7)	10.7 (8.2; 13.8)	2.3 (1.4; 3.9)	6.4 (4.6; 8.8)	12.9 (10.1; 16.2)
	3	Least	2.3 (1.3; 4.1)	4.5 (3.2; 6.4)	7.2 (5.4; 9.7)	2.8 (1.5; 5.5)	7.0 (4.7; 10.3)	11.3 (8.5; 14.9)
		Most	3.6 (2.1; 6.1)	6.8 (4.9; 9.5)	10.8 (8.1; 14.2)	4.8 (2.5; 9.0)	11.4 (7.8; 16.4)	17.9 (13.8; 22.9)
								
Major emergency treatment	0	Least	1.2 (0.7; 1.9)	4.1 (3.1; 5.3)	9.1 (7.3; 11.2)	1.2 (0.7; 2.1)	3.7 (2.7; 5.0)	8.1 (6.5; 10.0)
		Most	1.8 (1.2; 2.9)	6.2 (4.7; 8.0)	13.4 (10.9; 16.4)	2.1 (1.3; 3.5)	6.2 (4.6; 8.3)	13.1 (10.7; 16.0)
	3	Least	3.8 (2.1; 6.7)	7.9 (5.5; 11.2)	14.5 (10.8; 19.1)	2.6 (1.4; 4.9)	6.8 (4.7; 9.7)	11.6 (9.0; 14.8)
		Most	5.8 (3.3; 10.0)	11.7 (8.3; 16.3)	20.8 (15.9; 26.8)	4.3 (2.3; 8.0)	11.1 (7.8; 15.4)	18.3 (14.5; 22.8)
								
Major elective treatment	0	Least	0.4 (0.2; 0.6)	1.1 (0.9; 1.4)	2.6 (2.1; 3.2)	0.1 (0.1; 0.3)	0.5 (0.4; 0.8)	1.4 (1.0; 1.8)
		Most	0.6 (0.4; 0.9)	1.7 (1.3; 2.2)	4.0 (3.2; 5.0)	0.2 (0.1; 0.4)	0.9 (0.6; 1.3)	2.3 (1.8; 3.0)
	3	Least	1.4 (0.8; 2.4)	2.5 (1.7; 3.6)	4.9 (3.6; 6.6)	0.3 (0.1; 0.6)	1.0 (0.7; 1.6)	2.0 (1.5; 2.7)
		Most	2.1 (1.2; 3.7)	3.8 (2.7; 5.4)	7.4 (5.5; 9.9)	0.5 (0.2; 1.0)	1.7 (1.1; 2.6)	3.4 (2.5; 4.6)
**Stage 3**								
No major treatment	0	Least	3.2 (2.3; 4.5)	5.8 (4.6; 7.3)	9.8 (7.9; 12.1)	2.6 (1.7; 3.8)	4.7 (3.5; 6.2)	7.3 (5.8; 9.3)
		Most	4.9 (3.5; 6.9)	8.8 (6.9; 11.0)	14.5 (11.8; 17.7)	4.3 (2.9; 6.4)	7.7 (5.8; 10.2)	11.9 (9.4; 14.9)
	3	Least	6.1 (3.9; 9.4)	6.9 (5.2; 9.2)	9.9 (7.7; 12.7)	5.2 (3.0; 9.0)	8.5 (6.0; 11.9)	10.5 (8.0; 13.5)
		Most	9.2 (6.0; 13.8)	10.4 (7.9; 13.5)	14.6 (11.5; 18.4)	8.6 (5.1; 14.4)	13.7 (9.9; 18.7)	16.6 (13.0; 21.1)
								
								
Major emergency treatment	0	Least	2.0 (1.4; 2.9)	4.0 (3.1; 5.1)	8.1 (6.5; 10.1)	3.0 (2.1; 4.2)	5.7 (4.6; 7.2)	9.4 (7.8; 11.4)
		Most	3.1 (2.2; 4.4)	6.1 (4.8; 7.6)	12.0 (9.7; 14.8)	5.0 (3.5; 7.0)	9.4 (7.5; 11.7)	15.1 (12.5; 18.0)
	3	Least	6.3 (3.9; 10.1)	7.7 (5.5; 10.7)	13.0 (9.7; 17.3)	6.1 (3.6; 10.0)	10.3 (7.6; 13.9)	13.3 (10.5; 16.7)
		Most	9.4 (5.9; 14.8)	11.5 (8.3; 15.7)	18.8 (14.3; 24.4)	9.9 (6.0; 16.0)	16.4 (12.3; 21.5)	20.8 (16.8; 25.4)
								
Major elective treatment	0	Least	0.5 (0.4; 0.8)	0.9 (0.7; 1.2)	2.0 (1.5; 2.6)	0.3 (0.2; 0.6)	0.9 (0.6; 1.2)	1.6 (1.3; 2.2)
		Most	0.8 (0.6; 1.2)	1.4 (1.1; 1.9)	3.1 (2.4; 4.0)	0.6 (0.4; 0.9)	1.5 (1.1; 2.0)	2.8 (2.1; 3.7)
	3	Least	2.0 (1.2; 3.3)	2.1 (1.5; 3.0)	3.7 (2.6; 5.2)	0.7 (0.4; 1.4)	1.6 (1.1; 2.4)	2.4 (1.8; 3.3)
		Most	3.0 (1.8; 5.0)	3.2 (2.2; 4.6)	5.7 (4.1; 7.9)	1.2 (0.6; 2.3)	2.8 (1.9; 4.0)	4.1 (3.0; 5.5)
**Stage 4**								
No major treatment	0	Least	24.9 (22.7; 27.2)	33.2 (31.0; 35.4)	46.0 (43.4; 48.7)	24.4 (22.0; 27.0)	34.2 (31.8; 36.7)	49.1 (46.3; 51.9)
		Most	33.9 (31.2; 36.8)	43.5 (40.9; 46.1)	57.0 (54.2; 59.7)	35.6 (32.4; 38.9)	47.1 (44.2; 49.9)	62.3 (59.4; 65.0)
	3	Least	39.4 (31.6; 47.7)	37.5 (32.6; 42.6)	46.3 (41.8; 50.9)	40.5 (30.3; 51.6)	49.5 (42.9; 56.2)	58.8 (53.8; 63.6)
		Most	50.2 (41.8; 58.6)	48.2 (42.9; 53.4)	57.3 (52.7; 61.7)	53.8 (42.7; 64.5)	62.7 (56.3; 68.7)	70.9 (66.6; 74.9)
								
Major emergency treatment	0	Least	11.9 (9.8; 14.4)	18.1 (15.9; 20.6)	31.4 (27.9; 35.2)	11.1 (9.0; 13.6)	17.6 (15.3; 20.1)	29.6 (26.2; 33.2)
		Most	17.3 (14.5; 20.6)	25.6 (22.7; 28.7)	41.6 (37.3; 45.9)	17.5 (14.4; 21.2)	26.7 (23.5; 30.2)	41.8 (37.6; 46.1)
	3	Least	30.7 (22.1; 40.9)	30.9 (24.4; 38.2)	43.7 (36.3; 51.4)	20.8 (13.9; 29.9)	28.7 (23.0; 35.2)	38.3 (32.8; 44.1)
		Most	40.8 (30.6; 51.8)	41.0 (33.4; 48.9)	54.6 (46.9; 62.2)	31.0 (21.7; 42.1)	40.8 (33.9; 48.0)	51.5 (45.5; 57.5)
								
Major elective treatment	0	Least	3.8 (2.9; 5.0)	5.3 (4.4; 6.4)	10.4 (8.6; 12.6)	1.9 (1.3; 2.8)	4.1 (3.2; 5.2)	8.5 (6.9; 10.5)
		Most	5.8 (4.4; 7.5)	7.9 (6.6; 9.5)	15.3 (12.7; 18.3)	3.2 (2.2; 4.6)	6.8 (5.3; 8.5)	13.7 (11.2; 16.7)
	3	Least	12.7 (8.3; 19.0)	11.2 (8.2; 15.1)	18.1 (13.8; 23.4)	3.9 (2.2; 6.7)	7.4 (5.4; 10.2)	12.1 (9.4; 15.5)
		Most	18.5 (12.4; 26.6)	16.4 (12.3; 21.5)	25.6 (19.9; 32.2)	6.5 (3.7; 11.0)	12.1 (8.8; 16.2)	19.0 (15.0; 23.8)

Abbreviations: CI=confidence interval; PoD=probability of death.

**Table 3:** Average predicted probability of death within 90 days of colon cancer diagnosis: by deprivation

	**Males**		**Females**	
**Deprivation group**	**Average predicted probability**^a^ **(95% CI)**	**Average predicted probability, adjusted as if patients belonged to the deprivation group 1**^b^ **(95% CI)**	**Average predicted probability**^a^ **(95% CI)**	**Average predicted probability, adjusted as if patients belonged to the deprivation group 1**^b^ **(95% CI)**
Least deprived (1)	12.4 (11.8; 13.1)	12.4 (11.8; 13.1)	13.4 (12.7; 14.0)	13.4 (12.7; 14.0)
2	13.7 (13.0; 14.3)	12.9 (12.2; 13.5)	15.3 (14.7; 16.0)	14.1 (13.4; 14.8)
3	14.6 (13.9; 15.3)	13.3 (12.6; 14.0)	17.0 (16.3; 17.6)	14.8 (14.0; 15.5)
4	16.0 (15.3; 16.7)	13.6 (13.0; 14.3)	18.5 (17.8; 19.3)	15.5 (14.7; 16.2)
Most deprived (5)	17.7 (16.9; 18.6)	13.7 (13.0; 14.3)	19.9 (19.0; 20.7)	15.1 (14.4; 15.9)

Abbreviation: CI=confidence interval.

a Probability predicted for each deprivation group based on the observed value of the deprivation group, with the distribution of all prognostic factors remaining as observed.

b Probability predicted for each deprivation group adjusted to assume patients in each group belong to the least deprived group (group 1), with the distribution of all prognostic factors remaining as observed.
