# Supplementary figures and images for: Persistent inequalities in 90-day colon cancer mortality: an English cohort study
Source: Br J Cancer. 2017 Aug 31;117(9):1396–404. doi: 10.1038/bjc.2017.295 (PMC5672924; doi:10.1038/bjc.2017.295)

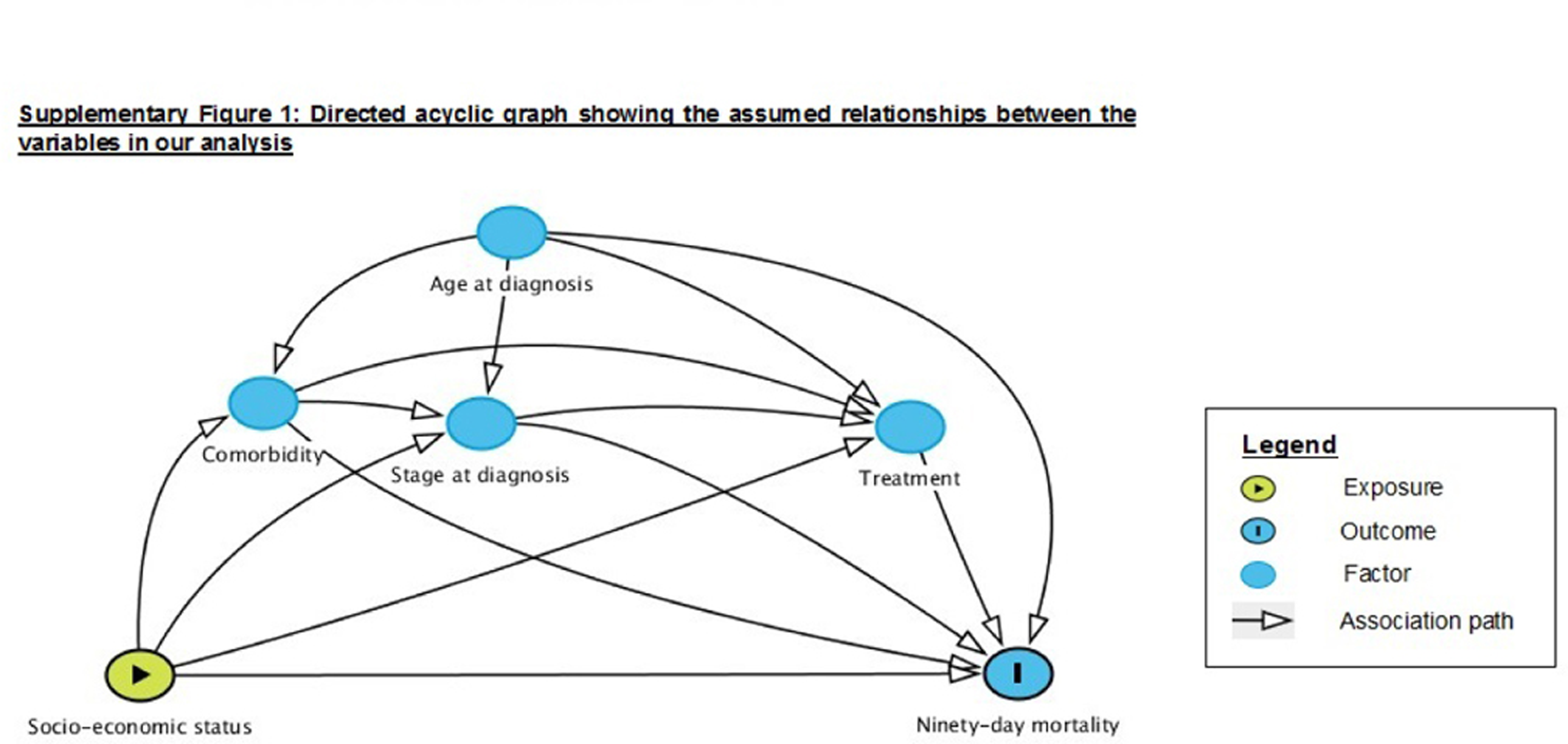

Supplement: Supplementary Figure 1 [file bjc2017295x1.tif]
