# Supplementary material for: Persistent inequalities in 90-day colon cancer mortality: an English cohort study
Source: Br J Cancer. 2017 Aug 31;117(9):1396–404. doi: 10.1038/bjc.2017.295 (PMC5672924; doi:10.1038/bjc.2017.295)
Supplement: Supplementary Figure 2 [file bjc2017295x2.pdf]

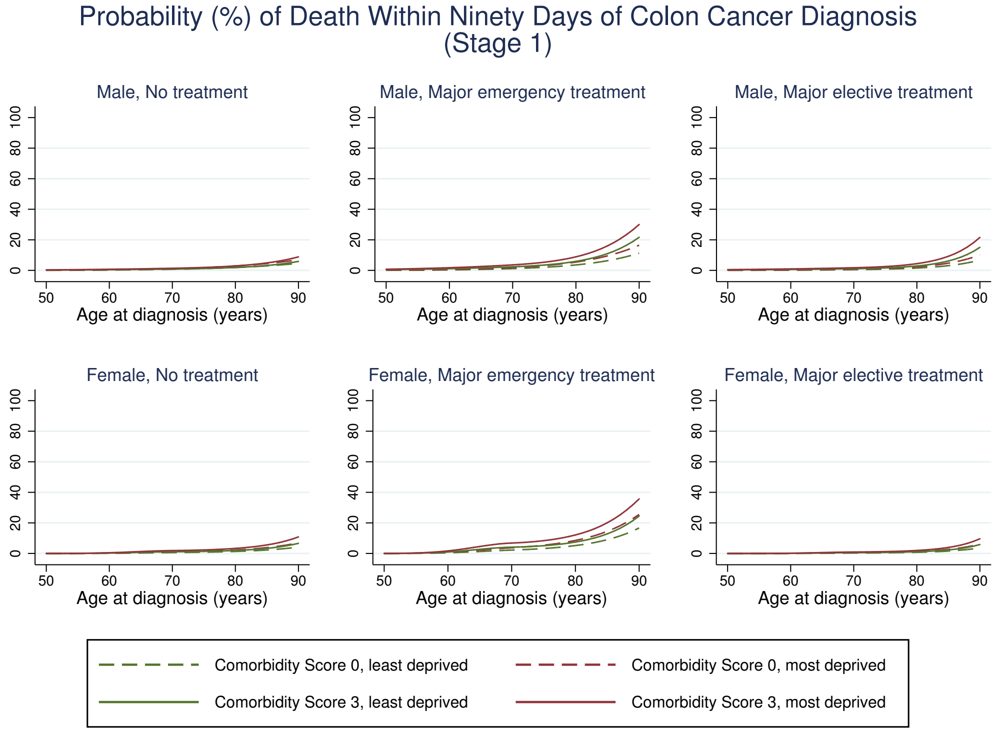
Probability (%) of Death Within Ninety Days of Colon Cancer Diagnosis (Stage 1)
Male, No treatment
Male, Major emergency treatment
Male, Major elective treatment
Female, No treatment
Female, Major emergency treatment
Female, Major elective treatment
0
20
40
60
80
100
50
60
70
80
90
Age at diagnosis (years)
Comorbidity Score 0, least deprived
Comorbidity Score 0, most deprived
Comorbidity Score 3, least deprived
Comorbidity Score 3, most deprived
