# Supplementary material for: Persistent inequalities in 90-day colon cancer mortality: an English cohort study
Source: Br J Cancer. 2017 Aug 31;117(9):1396–404. doi: 10.1038/bjc.2017.295 (PMC5672924; doi:10.1038/bjc.2017.295)
Supplement: Supplementary Legends [file bjc2017295x9.docx]

Supplementary Figure 1: Directed Acyclic Graph showing the assumed relationships between the variables in our analysis

Supplementary Figure 2: probability (%) of death within ninety days of colon cancer diagnosis in stage 1 patients according to age at diagnosis, among patients with (i) comorbidity score of 0 or 3 and (ii) from the least or the most deprived group.

Supplementary Figure 3: probability (%) of death within ninety days of colon cancer diagnosis in stage 3 patients according to age at diagnosis, among patients with (i) comorbidity score of 0 or 3 and (ii) from the least or the most deprived group.

Supplementary Figure 4: probability (%) of death within ninety days of colon cancer diagnosis in stage 4 patients according to age at diagnosis, among patients with (i) comorbidity score of 0 or 3 and (ii) from the least or the most deprived group.

Supplementary Table 1: Major surgery, as defined using OPCS-4 codes

Supplementary Table 2: Distribution of stage at diagnosis by treatment

Supplementary Table 3: Distribution of treatment by comorbidity score

Supplementary Table 4: Average predicted probability of death within ninety days of colon cancer diagnosis, by deprivation – additional analyses
